# Supplementary material for: PARP Inhibitors for Metastatic CRPC: More Answers than Questions, a Systematic Review and Meta-Analysis
Source: Pharmaceuticals (Basel). 2025 Jul 8;18(7):1015. doi: 10.3390/ph18071015 (PMC12298444; doi:10.3390/ph18071015)
Supplement: Supplementary file 1 [file pharmaceuticals-18-01015-s001.zip › pharmaceuticals-3693238-supplementary.pdf]

S1. Search Strategies

The search strategy was developed with the support of two expert epidemiologists with experience in systematic reviews (LI, SM). Electronic searches of registered databases were conducted until June 2023. Searches were conducted to identify randomised controlled trials examining PARP inhibitors in the treatment for metastatic castration-resistant prostate cancer. Medline (PubMed), Embase, CENTRAL (OVID) and ClinicalTrials were searched for clinical trials which met the predetermined inclusion criteria. Thematic experts provided feedback to the search strategy. We search also, abstracts and conferences of the American Society of Clinical Oncology (ASCO) and the European Society of Medical Oncology (ESMO) to identify relevant and ongoing clinical trials.

The strategies combined medical subject headings (MeSH) and keywords for the terms: "prostate cancer" AND "metastatic cancer" AND "nicotinamide adenine dinucleotide adenosine diphosphate ribosyltransferase inhibitor" OR "parp inhibitor".

Hand searches were performed to identify any studies not previously included.

N	Search	Reference
Medline (PubMed)		
1	'prostate cancer'[Title/Abstract] OR 'ca prostate'[Title/Abstract] OR 'cancer, prostate'[Title/Abstract] OR 'malignant prostate tumor'[Title/Abstract] OR 'malignant prostate tumour'[Title/Abstract] OR 'malignant prostatic tumor'[Title/Abstract] OR 'malignant prostatic tumour'[Title/Abstract] OR 'prostate malignancies'[Title/Abstract] OR 'prostate malignancy'[Title/Abstract] OR 'prostate malignant neoplasm'[Title/Abstract] OR 'prostate malignant tumor'[Title/Abstract] OR 'prostate malignant tumour'[Title/Abstract] OR 'prostatic cancer'[Title/Abstract] OR 'prostatic malignancies'[Title/Abstract] OR 'prostatic malignancy'[Title/Abstract]	161205
2	'nicotinamide adenine dinucleotide adenosine diphosphate ribosyltransferase inhibitor'[Title/Abstract] OR 'nad adp ribosyltransferase inhibitor'[Title/Abstract] OR 'parp inhibitor'[Title/Abstract] OR 'pars inhibitor'[Title/Abstract] OR 'poly adp ribose polymerase inhibitor'[Title/Abstract] OR 'poly adp ribose synthetase inhibitor'[Title/Abstract] OR 'olaparib'[Title/Abstract] OR 'talazoparib'[Title/Abstract] OR 'rucaparib'[Title/Abstract] OR 'niraparib'[Title/Abstract]	6759
3	1 and 2	648
4	3 Filters: Clinical Trial	59
Embase		
1	'prostate cancer'/exp OR 'ca prostate' OR 'cancer in the prostate' OR 'cancer of the prostate' OR 'cancer, prostate' OR 'malignancies of the prostate' OR 'malignancy of the prostate' OR 'malignant neoplasm of the prostate' OR 'malignant prostate tumor' OR 'malignant prostate tumour' OR 'malignant prostatic tumor' OR 'malignant prostatic tumour' OR 'malignant tumor of the prostate' OR 'prostate cancer' OR 'prostate gland cancer' OR 'prostate malignancies' OR 'prostate malignancy' OR 'prostate malignant neoplasm' OR 'prostate malignant tumor' OR 'prostate malignant tumour' OR 'prostatic cancer' OR 'prostatic malignancies' OR 'prostatic malignancy'	335661

2	'nicotinamide adenine dinucleotide adenosine diphosphate ribosyltransferase inhibitor'/exp OR 'nad adp ribosyltransferase inhibitor' OR 'parp inhibitor' OR 'pars inhibitor' OR 'nicotinamide adenine dinucleotide adenosine diphosphate ribosyltransferase inhibitor' OR 'poly (adp ribose) polymerase inhibitor' OR 'poly (adp ribose) polymerase inhibitors' OR 'poly (adp-ribose) polymerase inhibitors' OR 'poly adp ribose polymerase inhibitor' OR 'poly adp ribose synthetase inhibitor'	29215
3	1 AND 2	3479
4	1 AND 2 AND [randomized controlled trial]/lim	213
Cochrane Central Register of Controlled Trials - CENTRAL (OVID)		
1	('prostate cancer' or 'ca prostate' or 'cancer in the prostate' or 'cancer of the prostate' or 'cancer, prostate' or 'malignancies of the prostate' or 'malignancy of the prostate' or 'malignant neoplasm of the prostate' or 'malignant prostate tumor' or 'malignant prostate tumour' or 'malignant prostatic tumor' or 'malignant prostatic tumour' or 'malignant tumor of the prostate' or 'prostate cancer' or 'prostate malignancies' or 'prostate malignancy' or 'prostate malignant neoplasm' or 'prostate malignant tumor' or 'prostate malignant tumour' or 'prostatic cancer' or 'prostatic malignancies' or 'prostatic malignancy').ti,ab.	16183
2	('nicotinamide adenine dinucleotide adenosine diphosphate ribosyltransferase inhibitor' or 'nad adp ribosyltransferase inhibitor' or 'parp inhibitor' or 'pars inhibitor' or 'nicotinamide adenine dinucleotide adenosine diphosphate ribosyltransferase inhibitor' or 'poly adp ribose polymerase inhibitor' or 'poly adp ribose synthetase inhibitor' or 'olaparib' or 'talazoparib' or 'rucaparib' or 'niraparib').ti,ab.	1875
3	1 and 2	287
Clinical Trials		
1	Studies With Results | metastatic castration resistant prostate cancer | Phase 2, 3, 4, Not Applicable	180
2	olaparib OR Lynparza OR niraparib OR Zejula OR talazoparib OR rucaparib | castration resistant prostate cancer | Phase 2, 3, 4, Not Applicable	45
3	1 OR 2	225

S2. List of excluded studies

Excluded for not including subgroup information

- Clarke N, Armstrong A, Thiery-Vuillemin A, Oya M, Ye D, et al. PROPEL: A randomized phase III trial evaluating the efficacy and safety of olaparib combined with abiraterone as first-line therapy in patients with metastatic castration-resistant prostate cancer (mCRPC). European Urology, Supplements (2019) 18:1 (e1824). Date of Publication: 1 Mar 2019.

- Francis P, Fred S, Andrew A, Antoine T, Mototsugu O, et al. PROpel: Phase III trial of olaparib and abiraterone vs placebo and abiraterone as firstline (1L) therapy for patients (pts) with metastatic castration-resistant prostate cancer (mCRPC). Asia-Pacific Journal of Clinical Oncology. (2022) 18 Supplement 1 (80). Date of Publication: 1 Jul 2022.

- Machtens S, Augustin M, Feyerabend S, Hellmis E, Kretz T, et al. PROpel: A randomized, Phase III trial evaluating the efficacy and safety of olaparib combined with abiraterone as firstline therapy in patients with metastatic castration-resistant prostate cancer (mCRPC). Oncology Research and Treatment (2019) 42 Supplement 4 (287). Date of Publication: 1 Oct 2019.

- Saad F, Armstrong A, Thiery-Vuillemin A, Oya M, Loredo E, et al. PROpel: Phase III trial of olaparib (ola) and abiraterone (abi) versus placebo (pbo) and abi as first-line (1L) therapy for patients (pts) with metastatic castration-resistant prostate cancer (mCRPC). Journal of Clinical Oncology (2022) 40:6 SUPPL. Date of Publication: 2022.

Excluded for not including study outcomes

- Mateo J, Mossop H, Goodall J, Lorente D, Porta N, et al. Association of changes in circulating cell-free plasma DNA (cfDNA) and circulating tumor cells (CTC) during treatment with clinical outcome from olaparib in castration-resistant prostate cancer (CRPC): exploratory analyses from the TOPARP-A trial. Journal of clinical oncology. Conference:. Vol.35(6 Supplement 1) (no pagination):2017. Netherlands American Society of Clinical Oncology.

- Matsubara N, de Bono J, Olmos D, Procopio G, Kawakami S, et al. Olaparib Efficacy in Patients with Metastatic Castration-resistant Prostate Cancer and BRCA1, BRCA2, or ATM Alterations Identified by Testing Circulating Tumor DNA Clin Cancer Res. 2023 Jan 4;29(1):92-99. doi: 10.1158/1078-0432.CCR-21-3577.

- Roubaud G, Ozguroglu M, Penel N, Matsubara N, Mehra N, et al. Tolerability of olaparib (OLA) in patients (pts) with metastatic castration-resistant prostate cancer (mCRPC) and homologous recombination repair (HRR) gene alterations: PROfound. Annals of Oncology (2020) 31 Supplement 4 (S515-S516). Date of Publication: 1 Sep 2020.

- Roubaud G, Özgüroğlu M, Penel N, Matsubara N, Mehra N, et al. Olaparib tolerability and common adverse-event management in patients with metastatic castration-resistant prostate cancer: Further analyses from the PROfound study European Journal of Cancer (2022) 170 (73-84). Date of Publication: 1 Jul 2022.

- Saad F, Armstrong A, Thiery-Vuillemin A, Oya M, Shore N, et al. Prostate-specific antigen analyses in PROPEL: abiraterone and olaparib versus abiraterone and placebo as first-line therapy for metastatic castration-resistant prostate cancer. Journal of Urology (2023). 209 Supplement 4 (e131). Date of Publication: 1 Apr 2023.

Excluded for not being the study type

- Abida W, Campbell D, Patnaik A, Sautois B, Shapiro J, et al. Preliminary results from the TRITON2 study of rucaparib in patients (pts) with DNA damage repair (DDR)-deficient metastatic castration-resistant prostate cancer (mCRPC): Updated analyses. Annals of Oncology, 2019; 30, v327-v328.

- Abida W, Campbell D, Patnaik A, Shapiro J, Sautois B, et al. Non-BRCA DNA Damage Repair Gene Alterations and Response to the PARP Inhibitor Rucaparib in Metastatic Castration-Resistant Prostate Cancer: Analysis From the Phase II TRITON2 Study Rucaparib in mCRPC with a Non-BRCA DDR Gene Alteration. Clinical Cancer Research, 2020; 26(11), 2487-2496.

- Abida W, Patnaik A, Campbell D, Shapiro J, Bryce A, et al. Rucaparib in men with metastatic castration-resistant prostate cancer harboring a BRCA1 or BRCA2 gene alteration. Journal of Clinical Oncology, 2020; 38(32), 3763.

- Carreira S, Porta N, Arce-Gallego S, Seed G, Llop-Guevara A, et al. Biomarkers associating with PARP inhibitor benefit in prostate cancer in the TOPARP-B trial. Cancer Discovery (2021) 11:11 (2812-2827). Date of Publication: 1 Nov 2021.

- Clarke N, Wiechno P, Alekseev B, Sala N, Jones R, et al. Olaparib combined with abiraterone in patients with metastatic castration-resistant prostate cancer: a randomised, double-blind, placebo-controlled, phase 2 trial. The Lancet Oncology (2018) 19:7 (975-986). Date of Publication: 1 Jul 2018.

- de Bono J, Mehra N, Scagliotti G, Castro E, Dorff T, et al. Talazoparib monotherapy in metastatic castration-resistant prostate cancer with DNA repair alterations (TALAPRO-1): an open-label, phase 2 trial. Lancet oncology. Vol.22(9):1250-1264p, 2021. United Kingdom Lancet Publishing Group.

- de Bono J, Marcos E, Laird D, Fizazi K, Dorff T, et al. TALAPRO-1: Talazoparib monotherapy in metastatic castration-resistant prostate cancer (mCRPC) with DNA damage response alterations (DDRm)–Exploration of tumor genetics associated with prolonged benefit. Annals of Oncology, 2022; 33, S1167.

- Feyerabend S, Hussain M, Mateo J, Fizazi K, Saad F, et al. Profound: Phase III study of the efficacy and safety of olaparib versus enzalutamide or abiraterone acetate in men with metastatic castration-resistant prostate cancer (mCRPC) and homologous recombination repair gene (HRR) alterations. Oncology Research and Treatment (2020) 43 Supplement 1 (71-72). Date of Publication: 1 Feb 2020.

- Hussain M, Kocherginsky M, Agarwal N, Zhang J, Adra N, et al. BRCAAWAY: A randomized phase 2 trial of abiraterone, olaparib, or abiraterone + olaparib in patients with metastatic castration-resistant prostate cancer (mCRPC) with DNA repair defects. Journal of Clinical Oncology (2022) 40:16 Supplement 1. Date of Publication: 1 Jun 2022.

- Kaufman B, Shapira-Frommer R, Schmutzler RK, Audeh MW, Friedlander M, et al. Olaparib monotherapy in patients with advanced cancer and a germline BRCA1/2 mutation. J Clin Oncol. 2015 Jan 20;33(3):244-50. doi: 10.1200/JCO.2014.56.2728. Epub 2014 Nov 3.

- Mateo J. DNA-repair defects and olaparib in metastasic prostate cancer. Revista oncología. Issue 101, pp.50-51, 2015. pp.50-51.

- Mateo J, Porta N, Bianchini D, McGovern U, Elliott T, et al. Olaparib in patients with metastatic castration-resistant prostate cancer with DNA repair gene aberrations (TOPARP-B): a multicentre, open-label, randomised, phase 2 trial. The Lancet Oncology (2020) 21:1 (162-174). Date of Publication: 1 Jan 2020.

- Mateo J, Porta N, McGovern UB, Elliott T, Jones RJ, et al. TOPARP-B: A phase II randomized trial of the poly(ADP)-ribose polymerase (PARP) inhibitor olaparib for metastatic castration resistant prostate cancers (mCRPC) with DNA damage repair (DDR) alterations. Journal of Clinical Oncology (2019) 37 Supplement 15. Date of Publication: 1 May 2019.

- Mehra N, Fizazi K, de Bono JS, Barthélémy P, Dorff T, et al. Talazoparib, a Poly(ADP-ribose) Polymerase Inhibitor, for Metastatic Castration-resistant Prostate Cancer and DNA Damage Response Alterations: TALAPRO-1 Safety Analyses. Oncologist. 2022 Oct 1;27(10):e783-e795. doi: 10.1093/oncolo/oyac172.

- NCT01682772 TOPARP: A Phase II Trial of Olaparib in Patients With Advanced Castration Resistant Prostate Cancer. TOPARP.

- NCT02854436 An Efficacy and Safety Study of Niraparib in Men With Metastatic Castration-Resistant Prostate Cancer and DNA-Repair Anomalies. Galahad.

- NCT02952534 A Study of Rucaparib in Patients With Metastatic Castration-resistant Prostate Cancer and Homologous Recombination Gene Deficiency. TRITON2.

- NCT03148795 A Study of Talazoparib in Men With DNA Repair Defects and Metastatic Castration-Resistant Prostate Cancer. TALAPRO-1.

- Rao A, Heller G, Ryan C, VanderWeele D, Lewis L, et al. Alliance A031902 (CASPAR): A randomized, phase (ph) 3 trial of enzalutamide with rucaparib/placebo as novel therapy in first-line metastatic castration-resistant prostate cancer (mCRPC). Journal of Clinical Oncology (2022) 40:6 SUPPL. Date of Publication: 2022.

- Rao A, Ryan C, Weele D, Heller G, Lewis L, et. CASPAR (Alliance A031902): A randomized, phase III trial of enzalutamide (ENZ) with rucaparib (RUCA)/placebo (PBO) as a novel therapy in first-line metastatic castration-resistant prostate cancer (mCRPC). Journal of Clinical Oncology (2021) 39:6 SUPPL. Date of Publication: 1 Feb 2021

- Rodriguez-Vida A, Arranz Arija J, Daugaard G, Fizazi K, Gez E, et al. The TRITON clinical trial programme: evaluation of the PARP inhibitor rucaparib in patients with metastatic castration-resistant prostate cancer (mCRPC) associated with homologous recombination deficiency (HRD). European Urology, Supplements (2017) 16:10 (e2689-e2690). Date of Publication: 1 Nov 2017.

- Smith MR, Scher HI, Sandhu S, Efstathiou E, Lara PN Jr, et al. Niraparib in patients with metastatic castration-resistant prostate cancer and DNA repair gene defects (GALAHAD): a multicentre, open-label, phase 2 trial. Lancet Oncol. 2022 Mar; 23 (3): 362-373. doi: 10.1016/S1470-2045(21)00757-9. Epub 2022 Feb 4.

- Smith M, Sandhu S, Kelly W, Scher H, Efstathiou E, et al. Pre-specified interim analysis of GALAHAD: A phase II study of niraparib in patients (pts) with metastatic castration-resistant prostate cancer (mCRPC) and biallelic DNA-repair gene defects (DRD). Annals of Oncology, 2019; 30, v884-v885.

Does not provide additional information

- Agarwal N, Azad A, Shore ND, Carles J, Fay AP, et al. Plain language summary of the design of the TALAPRO-2 study comparing talazoparib and enzalutamide versus enzalutamide and placebo in men with metastatic castration-resistant prostate cancer. Future oncology (London, England). 2022. Vol.18(27):2979-2986p. United Kingdom.

- Agarwal N, Azad A, Shore N, Carles J, Fay A, et al. TALAPRO-2: A phase 3 randomized study of enzalutamide (ENZA) plus talazoparib (TALA) versus placebo in patients with new metastatic castration-resistant prostate cancer (mCRPC). Journal of Clinical Oncology. (2021) 39:15 SUPPL.

- Agarwal N, Shore ND, Dunshee C, Karsh LI, Azad A, et al. TALAPRO-2: A placebo-controlled phase III study of talazoparib (TALA) plus enzalutamide (ENZA) for patients with first-line metastatic castration-resistant prostate cancer (mCRPC). Journal of Clinical Oncology. (2020), 38:6 Supplement. Date of Publication: 2020.

- Agarwal N, Shore ND, Dunshee C, Karsh LI, Sullivan B, et al. TALAPRO-2: A two-part, placebo-controlled phase III study of talazoparib (TALA) with enzalutamide (ENZA) in metastatic castration-resistant prostate cancer (mCRPC). Journal of Clinical Oncology. (2019), 37 Supplement 7. Date of Publication: 1 Mar 2019.

- Agarwal N, Shore ND, Dunshee C, Karsh LI, Sullivan B, et al. Clinical and safety outcomes of TALAPRO-2: A two-part phase III study of talazoparib (TALA) in combination with enzalutamide (ENZA) in metastatic castration-resistant prostate cancer (mCRPC). Journal of Clinical Oncology. (2019), 37 Supplement 15. Date of Publication: 1 May 2019.

- Agarwal N, Shore ND, Dunshee C, Karsh LI, Sullivan B, et al. TALAPRO-2: Part 2 (P2) of the placebo-controlled phase 3 study of talazoparib (TALA) with enzalutamide (ENZA) in metastatic castration-resistant prostate cancer (mCRPC). Journal of Clinical Oncology. (2019), 37 Supplement 15. Date of Publication: 1 May 2019.

- Bryce AH, Piulats JM, Reaume MN, Ostler PJ, McDermott RS, et al. Rucaparib for metastatic castration-resistant prostate cancer (mCRPC): TRITON3 interim overall survival and efficacy of rucaparib vs docetaxel or second-generation androgen pathway inhibitor therapy. Journal of Clinical Oncology. (2023), 41:6 Supplement (18). Date of Publication: 2023.

- Chi KN, Rathkopf D, Smith MR, Efstathiou E, Attard G, et al. Niraparib and Abiraterone Acetate for Metastatic Castration-Resistant Prostate Cancer. Journal of clinical oncology : official journal of

the American Society of Clinical Oncology. (2023), 41:18 (3339-3351) Date of Publication: 20 Jun 2023.

- Chi KN, Rathkopf DE, Smith MR, Efstathiou E, Attard G, et al. Phase 3 MAGNITUDE study: First results of niraparib (NIRA) with abiraterone acetate and prednisone (AAP) as first-line therapy in patients (pts) with metastatic castration-resistant prostate cancer (mCRPC) with and without homologous recombination repair (HRR) gene alterations. Journal of Clinical Oncology. (2022), 40:6 SUPPL Date of Publication: 2022.

- Chi KN, Rathkopf D, Smith MR, Efstathiou E, Attard G, et al. Niraparib and Abiraterone Acetate for Metastatic Castration-Resistant Prostate Cancer.
- Clarke NW, Armstrong AJ, Thiery-Vuillemin A, Oya M, Shore N, et al. PROpel: Efficacy of abiraterone + olaparib vs abiraterone + placebo in the first-line treatment of patients with asymptomatic/mildly symptomatic metastatic castration-resistant prostate cancer (mCRPC) at baseline. European Urology. (2023) 83 Supplement 1 (S1674-S1675) Date of Publication: 1 Feb 2023

- Clarke NW, Armstrong AJ, Thiery-Vuillemin A, Oya M, Shore ND, et al. Final overall survival (OS) in PROpel: Abiraterone (abi) and olaparib (ola) versus abiraterone and placebo (pbo) as first-line (1L) therapy for metastatic castration-resistant prostate cancer (mCRPC). Journal of Clinical Oncology. (2023), 41:6 Supplement (LBA16) Date of Publication: 2023.

- De Bono JS, Fizazi K, Saad F, Shore ND, Roubaud G, et al. PROfound: Efficacy of olaparib (ola) by prior taxane use in patients (pts) with metastatic castration-resistant prostate cancer (mCRPC) and homologous recombination repair (HRR) gene alterations. Journal of Clinical Oncology. (2020), 38:6 Supplement. Date of Publication: 2020.

- de Bono JS, Mateo J, Fizazi K, Saad F, Shore N, et al. Final overall survival (OS) analysis of PROfound: Olaparib vs physician's choice of enzalutamide or abiraterone in patients (pts) with metastatic castration-resistant prostate cancer (mCRPC) and homologous recombination repair (HRR) gene alterations. Annals of Oncology. (2020), 31 Supplement 4 (S508). Date of Publication: 1 Sep 2020.

- De Bono JS, Matsubara N, Penel N, Mehra N, Kolinsky MP, et al. Exploratory gene-by-gene analysis of olaparib in patients (pts) with metastatic castrationresistant prostate cancer (mCRPC): PROfound. Journal of Clinical Oncology. (2021), 39:6 SUPPL. Date of Publication: 1 Feb 2021.

- Efstathiou E, Smith MR, Sandhu S, et al. Niraparib with abiraterone acetate and prednisone in patients with metastatic castration-resistant prostate cancer and homologous recombination repair gene alterations: second interim analysis (IA2) of MAGNITUDE. J Clin Oncol. 2023;41(suppl 6):170. doi:101200/JCO2023416_suppl170

- Efstathiou E, Smith MR, Sandhu S, Attard G, Saad M, et al. Niraparib (NIRA) with abiraterone acetate and prednisone (AAP) in patients (pts) with metastatic castration-resistant prostate cancer (mCRPC) and homologous recombination repair (HRR) gene alterations: Second interim analysis (IA2) of MAGNITUDE. Journal of Clinical Oncology. (2023), 41:6 Supplement (170). Date of Publication: 2023.

- Hussain M, Mateo J, Fizazi K, Saad F, Shore N, et al. Survival with Olaparib in Metastatic Castration-Resistant Prostate Cancer. New England journal of medicine. Vol.383(24):2345-2357p, 2020.

- Hussain M, Mateo J, Fizazi K, Saad F, Shore N, et al. PRO found: Phase III study of olaparib versus enzalutamide or abiraterone for metastatic castration-resistant prostate cancer (mCRPC) with homologous recombination repair (HRR) gene alterations Annals of Oncology (2019) 30 Supplement 5 (v881-v882). Date of Publication: 1 Oct 2019.

- Matsubara N, Nishimura K, Kawakami S, Joung JY, Uemura H, et al. Olaparib in patients with mCRPC with homologous recombination repair gene alterations: pROfound Asian subset analysis. Japanese journal of clinical oncology. Vol.52(5):441-448p, 2022. United Kingdom NLM (Medline).

- NCT02987543 Study of Olaparib (Lynparza™) Versus Enzalutamide or Abiraterone Acetate in Men With Metastatic Castration-Resistant Prostate Cancer (PROfound Study). PROfound.

- NCT03748641 A Study of Niraparib in Combination With Abiraterone Acetate and Prednisone Versus Abiraterone Acetate and Prednisone for Treatment of Participants With Metastatic Prostate Cancer. MAGNITUDE.

- Oya M, Armstrong AJ, Thiery-Vuillemin A, Shore N, Procopio G, et al. Biomarker analysis and updated results from the phase III PROpel trial of abiraterone (abi) and olaparib (ola) vs abi and placebo (pbo) as first-line (1L) therapy for patients (pts) with metastatic castration-resistant prostate cancer (mCRPC). Annals of Oncology. (2022), 33 Supplement 9 (S1495). Date of Publication: 1 Nov 2022.

- Sandhu S, Attard G, Olmos D, Efstathiou E, Castro E, et al. Gene-by-gene analysis in the MAGNITUDE study of niraparib (NIRA) with abiraterone acetate and prednisone (AAP) in patients (pts) with metastatic castration-resistant prostate cancer (mCRPC) and homologous recombination repair (HRR) gene alterations. Journal of Clinical Oncology. (2022), 40:16 Supplement 1. Date of Publication: 1 Jun 2022.

- Sandhu SK, Hussain M, Mateo J, Fizazi K, Saad F, et al. PROfound: Phase III study of olaparib versus enzalutamide or abiraterone for metastatic castration-resistant prostate cancer (mCRPC) with homologous recombination repair (HRR) gene alterations. Annals of Oncology (2019) 30 Supplement 9 (ix188-ix189). Date of Publication: 1 Nov 2019.

- Thiery-Vuillemin A, Saad F, Armstrong AJ, Oya M, Vianna KCM, et al. Tolerability of abiraterone (abi) combined with olaparib (ola) in patients (pts) with metastatic castration-resistant prostate cancer (mCRPC): Further results from the phase III PROpel trial. Journal of Clinical Oncology. (2022), 40:16 Supplement 1. Date of Publication: 1 Jun 2022.

- A Phase 3 Study of Niraparib in Combination with Abiraterone Acetate and Prednisone Versus Abiraterone Acetate and Prednisone for Treatment of Patients with Metastatic Prostate Cancer. 2019. [No additional source data available.]

- A Study of Niraparib in Combination With Abiraterone Acetate and Prednisone Versus Abiraterone Acetate and Prednisone for Treatment of Participants With Metastatic Prostate Cancer. 2018. [No additional source data available.]

- Study on Olaparib Plus Abiraterone as First-line Therapy in Men With Metastatic Castration-resistant Prostate Cancer. 2018. [No additional source data available.]
